# First record of *Baylisascaris procyonis* in the wild invasive Northern raccoon (*Procyon lotor*) in the Czech Republic

**DOI:** 10.3389/fvets.2025.1686564

**Published:** 2025-10-14

**Authors:** Michal Benovics, Lívia Švantnerová, Ondřej Mikulka, Lucie Škorpíková, Vlastimil Skoták, Jan Cukor

**Affiliations:** ^1^Department of Zoology, Faculty of Natural Sciences, Comenius University in Bratislava, Bratislava, Slovakia; ^2^Unit for Environmental Sciences and Management, North-West University, Potchefstroom, South Africa; ^3^Faculty of Forestry and Wood Technology, Mendel University in Brno, Brno, Czechia; ^4^Forestry and Game Management Research Institute, Jíloviště, Czechia; ^5^Department of Botany and Zoology, Faculty of Science, Masaryk University, Brno, Czechia; ^6^Faculty of Forestry and Wood Sciences, Czech University of Life Sciences Prague, Prague, Czechia

**Keywords:** introductions, zoonoses, Nematoda, neurotropic diseases, Doupov Highlands, molecular identification

## Abstract

*Baylisascaris procyonis*, a zoonotic nematode originated in North America, is a significant cause of *larva migrans* in humans and wildlife. Here we report the first confirmed record of *B. procyonis* in free-ranging invasive Northern raccoons (*Procyon lotor*) in the Czech Republic, based on combined morphological and molecular evidence. 10 raccoons were examined from the Doupov Highlands subpopulation between 2023 and 2025, *B. procyonis* were recovered from three individuals. Morphological and molecular analyses of ITS and COI regions confirmed the species identity, showing 99.3%−100% similarity to known conspecific sequences. This finding extends the recognized range of this zoonotic parasite in Central Europe, underscoring the potential public health risks posed by environmental contamination with infective eggs. Our results highlight the need for ongoing surveillance of invasive raccoon populations to monitor the spread of this pathogenic zoonotic parasites.

## Introduction

Biological invasions pose a global challenge for biodiversity conservation, causing a wide range of negative impacts on native ecosystems and their components (1). The introduction of invasive species is typically driven by multiple factors, including propagule pressure associated with international trade and the ecological condition of the new environment (2). One of the most thoroughly researched and consistently confirmed impacts of biological invasions is the disturbance of the environment for native, particularly protected species, resulting in the destabilization of specific habitats. In addition to ecological effects, invasive species may pose direct risks to human health.

This is best exemplified by the Northern raccoon (*Procyon lotor*), whose native range is in North America, but whose population has been rapidly increasing in Central Europe in recent decades. The European raccoon population is currently considered to be beyond effective control, with Germany as the core distribution area. At the same time the species rapidly expands into other countries such as Poland, Austria, France, and the Czech Republic (3). Along with raccoons, parasites associated with this species—and for which it serves as a suitable host—could also be introduced into Europe. One such common parasite in the native distribution range is *Baylisascaris procyonis*, whose occurrence has been relatively well documented in North America (4–6) and its presence and potential spread in Europe is apparent from the recent reports from Germany (7–9), Poland (10), Denmark (11), Norway (12), Austria (13), and Italy (14).

*Baylisascaris procyonis* is an intestinal nematode of raccoons, causing severe or fatal *larva migrans* syndrome. Over 150 species of birds and mammals (4) are known to be susceptible to infection, including humans (4, 5). Humans serve as paratenic hosts and infection occurs through ingestion of embryonated eggs from contaminated environments, often in urban or peri-urban areas (15, 16). The larvae are neurotropic and can migrate to the central nervous system, leading to eosinophilic meningoencephalitis, especially in young children or immunocompromised individuals (17, 18). It is also recognized as a cause of ocular disease in humans. In contrast to neural *larva migrans* (NLM), which is primarily restricted to infants and young children, isolated ocular *larva migrans* (OLM) usually occurs in otherwise healthy adults (19, 20). Due to its environmental persistence and high pathogenicity, *B. procyonis* is considered an important emerging zoonosis. To date, more than 50 human cases of infection have been confirmed worldwide (21), most of them from the USA, where at least 35 cases have been reported (22). Data from Europe are very scarce. Only two non-fatal clinical cases have been described in the literature (23). In addition to these clinical cases, four individuals with serologically confirmed exposure were reported in Germany (24). Our research therefore aimed to investigate the parasite fauna of raccoons in Czechia, with a special focus on a zoonotic *B. procyonis*.

## Materials and methods

### Material collection and identification

**Figure 1:**
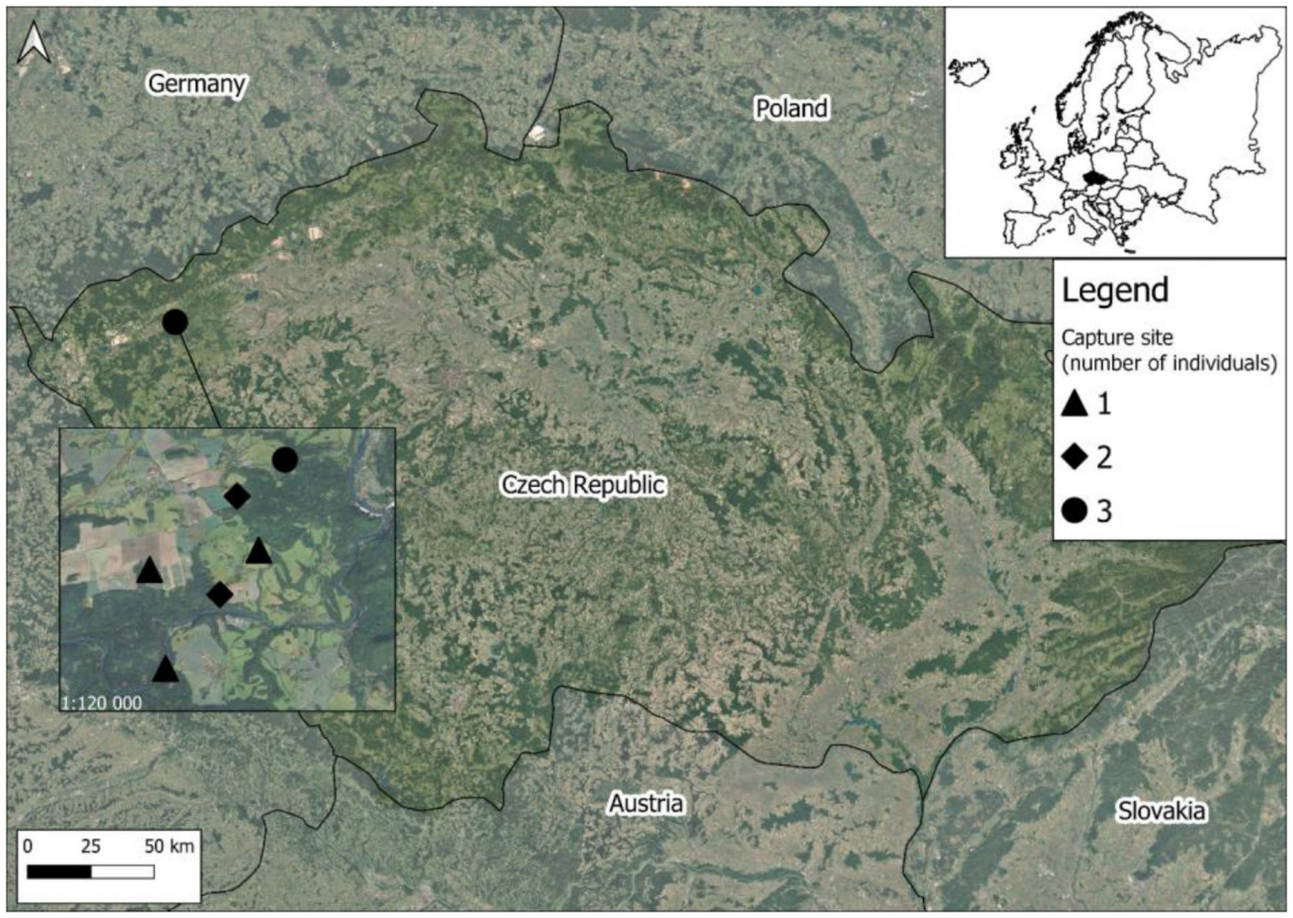
Location of the Doupov Highlands and respective sites where infected raccoons were collected.

Ten raccoons were collected between 2023 and 2025 from the Doupov Highlands (Doupovské Hory, Figure 1) subpopulation, an area characterized by mixed forests, semi-natural broadleaved stands, and open habitats within a military training area. The animals were collected through hunting, a standard wildlife management practice. According to Decree No. 454/2021 Coll. on the designation of invasive species requiring regulation, raccoons are hunted by any hunter holding a hunting license issued by the manager of the respective hunting ground. They were placed in plastic bags, then labeled with information on the sampling location, sex, and body weight. The samples were transported to the laboratory, where the animals were dissected and the intestines and remaining body parts were frozen separately. Before parasitological examination, the intestines were gradually thawed and longitudinally incised. Macroscopic observation revealed the presence of white-yellowish ascarid nematodes in the small intestine of three animals. These were removed, washed and stored in 70% ethanol for subsequent morphological and molecular evaluation. The intestines were examined under a stereomicroscope, and the contents were cleaned using the sieve system [following (25)]. Any additional helminths identified were also preserved in either 70 or 96% ethanol for future studies focusing on the parasite diversity of raccoons in Czechia. The identification of the collected ascarid nematodes followed the guidelines provided by (17, 26, 27). The specimens were then cut into three parts, with the middle section designated for subsequent molecular analyses.

### Molecular and phylogenetic analyses

Genomic DNA was extracted from cut sections of six nematodes using the DNeasy Blood & Tissue Kit (Qiagen, Hilden, Germany) following the manufacturer's protocol. Two genomic regions were amplified: the ITS1-5.8S-ITS2 ribosomal regions using primers 18SNemF and 26SNemR (28) and the partial mitochondrial COI gene with primers CO1-F and CO1-R (29). The PCR reactions were performed in a 20 μl reaction mixture containing 14 μl nuclease-free water, 4 μl FIREPol Master Mix Ready to Load (Solis BioDyne, Tartu, Estonia), 0.5 μM of each primer, and 1 μl of DNA template. PCR products were detected by electrophoresis in 1% agarose gels stained with GoodView (SBS Genetech, Bratislava, Slovakia). The thermocycler conditions for amplification were retrieved from (30), and resulting amplicons were subjected to Sanger sequencing in Macrogen Europe (Amsterdam, Netherlands) using the PCR primers.

To confirm the species of the collected ascarid nematodes and their phylogenetic relationships to other Ascarididae, additional orthologous COI sequences from congeners or other phylogenetically close species were retrieved from GenBank (accession numbers are included within phylogenetic trees). Three *Toxocara* spp. ortholog sequences were used as an outgroup for rooting the phylogenetic tree according to (30). The sequences were aligned with the fast Fourier transform algorithm, implemented in MAFFT (31), using the G-INS-i refinement method. The data were treated as codon partitioned, and a general time-reversible model [GTR (32)] was selected independently for each position within the codon, including both a gamma distribution and the proportion of invariable sites. Phylogenetic trees were constructed utilizing Bayesian inference (BI) and maximum likelihood (ML) approaches in MrBayes 3.2. (33) and RAxML 8.1.12 (34, 35), respectively. The BI analysis was run for 2 × 10^6^ generations, sampling trees every 100 generations. The initial 30% of all saved trees were discarded as “burn-in” after checking that the standard deviation split frequency fell below 0.01. The convergence of the runs and the parameters of individual runs were checked using Tracer v. 1.7.1 (36). Posterior probabilities for each tree node were calculated as the frequency of samples recovering a given clade. The clade bootstrap support for ML trees was assessed by simulating 10^3^ pseudoreplicates.

## Results

**Figure 2:**
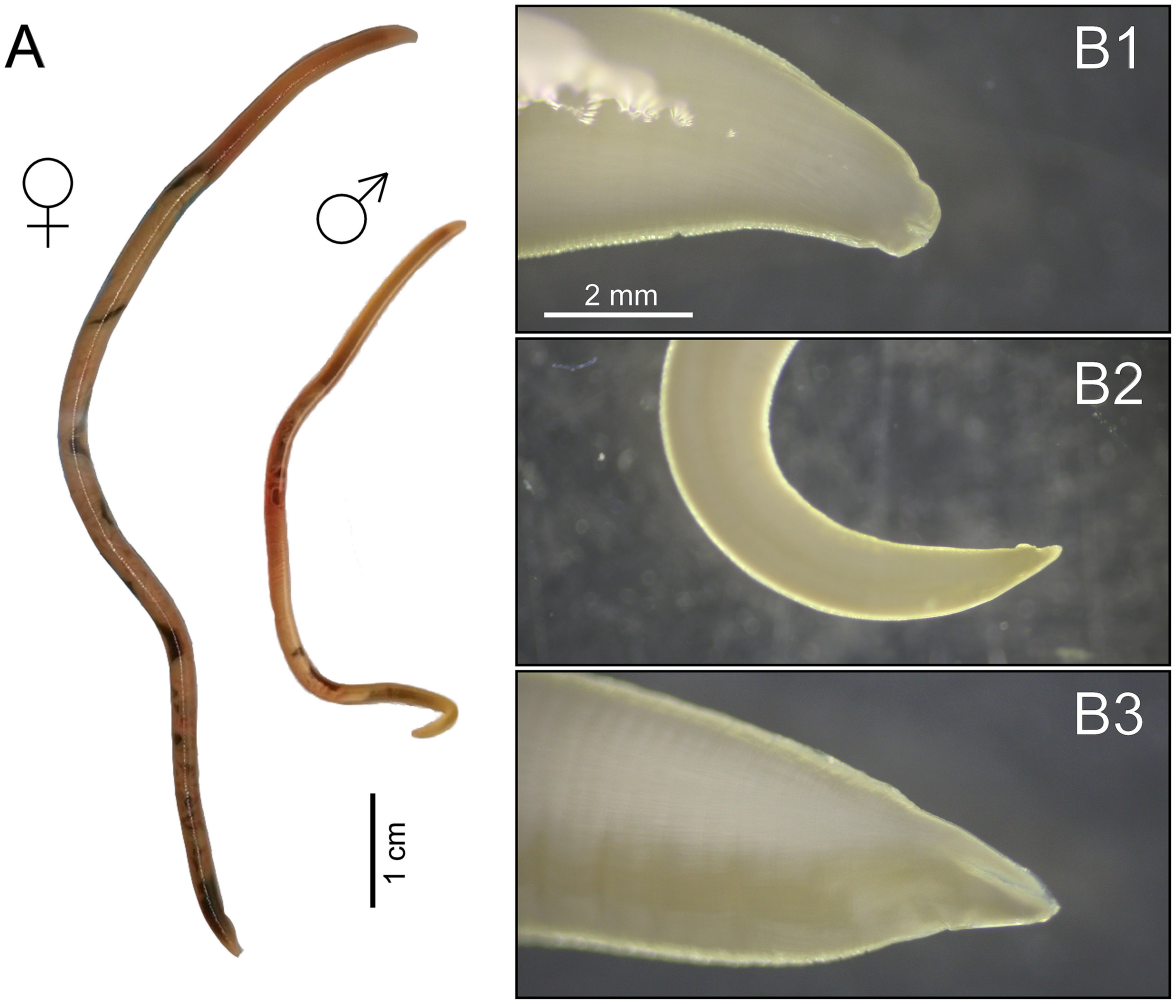
*Baylisascaris procyonis* photo comparison of female and male **(A)**; and microphotographs of morphological details: anterior end with typical lips **(B1)**; male posterior end with tip of spicule **(B2)**; and female posterior end **(B3)**.

**Figure 3:**
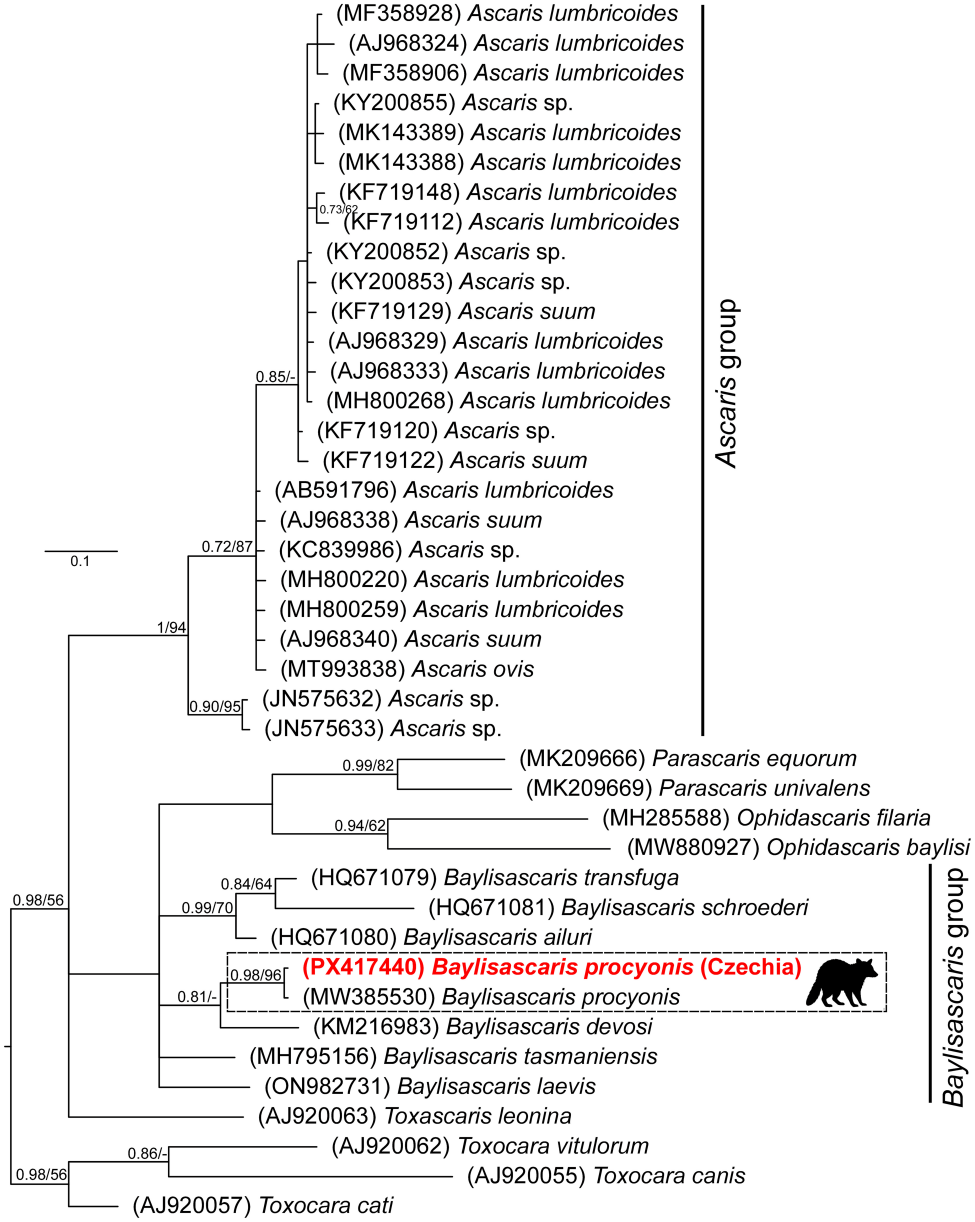
The phylogenetic tree of the Ascarididae species was built from partial COI sequences using Bayesian inference. The tree is rooted using *Toxocara* spp. as an outgroup. Values at the nodes indicate posterior probabilities from BI and bootstrap values from ML analyses. Dashes indicate nodal support values below 0.70 and 50, respectively. The newly obtained sequence from Czechia is in red.

A total of one, two, and eight ascarid nematodes were collected from each infected raccoon. Morphological examinations of all 11 ascarids (Figure 2) revealed characteristics consistent with *B. procyonis* as reviewed by (26). The majority of the collected specimens were females (F:M ratio 8:3). The length of the collected specimens ranged from 4.2 to 10.1 cm, and the width from 1 to 3 mm. All specimens carried identical ITS1-5.8S-ITS2 and COI alleles, respectively. The newly obtained ITS1-5.8S-ITS2 sequence (1,045 bp long, GenBank accession number PX417439) showed 99.3–100% similarity to conspecific sequences in GenBank (MZ092850-MZ092855) obtained from raccoons in China. The final COI alignment for building phylogenetic trees included 41 taxa and spanned 383 unambiguously aligned nucleotide positions. Both BI and ML analyses generated trees with identical topologies. The BI tree with posterior probabilities and bootstrap values (corresponding to the ML tree) along respective nodes is presented in Figure 3. The resulting trees clearly showed that the newly obtained COI sequences from Czechia (PX417440) are identical to the representative conspecific sequence retrieved from GenBank (MW385530, originating from a USA specimen). Congruently with previous studies, the *Baylisascaris* species showed a paraphyletic grouping with *Parascaris* and *Ophidascaris* species.

## Discussion

This study represents the first morphologically and molecularly confirmed detection of *Baylisascaris procyonis* in wild raccoons within the Czech Republic, extending the known range of this zoonotic nematode in Central Europe. Although Tenora and Staněk (37) previously reported this species from the vicinity of Brno based on larvae morphology, the origin of the host raccoons was uncertain, and the identification of nematodes was not confirmed by molecular data. Our findings thus provide the first definitive evidence of *B. procyonis* established in this region. Although only 3 out of 10 examined raccoons in Doupov Highlands were infected with *B. procyonis*, this finding is potentially epidemiologically significant given the parasite's capacity to cause severe disease in humans and its increasing prevalence in raccoon populations throughout Europe. In two of the three parasitized raccoons, the *B. procyonis* specimens were congruent in size with previously recorded conspecifics; i.e., males measuring 6.3 and 6.8 cm, and a single female measuring 10.1 cm [see reviewed meristic data in (14) and (38)]. In contrast, the third raccoon harbored eight roundworms that were comparatively smaller than previously reported, all females measuring 4.2–5.0 cm. The absence of eggs and the presence of only a weakly developed uterus suggest that these specimens were in the subadult stage explaining smaller size.

In neighboring countries, this parasite was recorded with a remarkably high prevalence in Germany, where out of 234 examined raccoons, 95% were carrying *B. procyonis* (39). In Austria, near the German and Czech borders, one raccoon found dead was parasitized by *B. procyonis* (13). As the molecular analyses showed the origin of this animal in Germany, this finding further supports the eastward spread of raccoons harboring allochthonous zoonotic diseases. A similar hypothesis was also postulated by Polish authors (10) as the parasite species were coprologically confirmed in raccoons near the Poland-Germany border.

The high prevalence of *B. procyonis* in some parts of Europe may be attributed to reduced parasite diversity in introduced European raccoon populations and high local host densities [such as in Germany (40)], which facilitate the transmission of diseases. Considering the direct life cycle of the parasite and its environmental contamination with infective eggs via feces, these parasites can easily be transmitted to humans, especially in areas where raccoons are expanding into human settlements (15, 41). Moreover, the higher prevalence of *B. procyonis* could also be related to Northern raccoon population density, considering the population of raccoons in Germany is approximately eight times bigger than in Czechia, as can be estimated from the harvest numbers obtained in the same hunting season (42, 43).

The detection of *B. procyonis* in the Czech Republic represents a significant finding in the context of invasive parasite monitoring and public health risk. This report from the western part of the country confirms the continued expansion of *B. procyonis* beyond its original introduced range in Germany and into neighboring countries. Given the parasite's zoonotic potential and its capacity to cause severe neurological disease in humans, its presence in the Czech Republic raises concerns about environmental contamination and the potential risk to both wildlife and human populations. The finding underscores the importance of continued surveillance of invasive raccoon populations, particularly synanthropic populations, as they often accommodate multiple zoonotic parasite species (39). Therefore, the necessity of eradicating the northern raccoon, an invasive species with potentially substantial impacts on native ecosystems, is further underscored from a public health perspective given the risk of parasite transmission. However, the management strategies aimed at controlling raccoon population size and dispersal potential have failed so far (44) highlighting the need for further research into population control measures.

## Data Availability

The raw data supporting the conclusions of this article will be made available by the authors, without undue reservation. Newly generated sequence data are available in GenBank under accession numbers PX417439 and PX417440.
